# Taste Perception and Caffeine Consumption: An fMRI Study

**DOI:** 10.3390/nu11010034

**Published:** 2018-12-24

**Authors:** Laura Gramling, Eleni Kapoulea, Claire Murphy

**Affiliations:** 1 Department of Psychology, San Diego State University, San Diego, CA 92182, USA; lauragramling@gmail.com (L.G.); ekapoulea@sdsu.edu (E.K.); 2 San Diego State University/University of California, San Diego Joint Doctoral Program, San Diego, CA 92120, USA; 3 Department of Psychiatry, University of California, San Diego, CA 92093, USA

**Keywords:** fMRI, caffeine, taste, memory

## Abstract

Caffeine is ubiquitous, yet its impact on central taste processing is not well understood. Although there has been considerable research on caffeine’s physiological and cognitive effects, there is a paucity of research investigating the effects of caffeine on taste. Here we used functional magnetic resonance imaging (fMRI) to investigate group differences between caffeine consumers and non-consumers in blood-oxygenation-level-dependent (BOLD) activation during hedonic evaluation of taste. We scanned 14 caffeine consumers and 14 caffeine non-consumers at 3 Tesla, while they rated three tastes: caffeine (bitter), sucrose (sweet), and saccharin (sweet with bitter after taste), in aqueous solutions. Differences in BOLD activation were analyzed using voxel wise independent samples *t*-tests within Analysis of Functional Neuroimage (AFNI). Results indicated that during the hedonic evaluation of caffeine or sucrose, caffeine non-consumers had significantly greater activation in neuronal areas associated with memory and reward. During the hedonic evaluation of saccharin, caffeine consumers had significantly greater activation in areas associated with memory and information processing. The findings suggest caffeine consumption is associated with differential activation in neuronal areas involved in reward, memory, and information processing. Further research on intensity and hedonics of bitter and sweet stimuli in caffeine consumers and non-consumers will be of great interest to better understand the nature of differences in taste perception between caffeine consumers and non-consumers.

## 1. Introduction

Caffeine consumption is ubiquitous. It currently ranks as the most popular psychostimulant in the world [1]. Eighty-five percent of the United States’ population consumes at least one caffeinated beverage daily [2]. Many beverages contain caffeine, including coffee, the most widely consumed beverage after water [3]. Other widely consumed caffeinated beverages are tea and energy drinks, which typically contain a high caffeine content, as well as a high glucose content [2,4]. Despite caffeine’s bitter taste and the fact that bitter tastes often discourage intake, coffee and tea remain two of the most widely ingested beverages [5]. Caffeine’s widespread consumption warrants a better understanding of its effects.

Evidence supporting caffeine’s ability to exert beneficial effects is abundant [6]. When consumed in moderate amounts, caffeine has been reported to decrease fatigue and increase energy [6]. Caffeine has also been reported to increase motor performance on sustained response tasks. For example, participants randomly assigned in a double-blind study to either consume a drink containing 40 mg of caffeine or placebo, showed enhanced performance on a selective attention task when exposed to the experimental condition [7]. Further, caffeine produces mild autonomic nervous system arousal and improved mood when compared to a non-caffeinated placebo [8]. During a visuomotor task, participants demonstrated increased blood-oxygenation-level-dependent (BOLD) activation in the putamen and insula after consuming 200 mg of caffeine [9]. The putamen is part of the basal ganglia, an area that has been shown to modulate the top-down influence of the prefrontal cortex on sensory processing in humans [10]. Increased activation in the striatum following caffeine consumption suggests that caffeine can act as a cognitive enhancer by modulating these attentional areas [9].

While caffeine consumed at moderate doses may provide consumers with a number of favorable effects, research suggests negative consequences as a result of caffeine consumed at higher doses [8,11]. Increasing caffeine consumption can exert dose-dependent effects on a number of acute autonomic responses, including increased blood pressure [8]. Caffeine consumed at 300–800 mg can induce anxiety, nervousness, and insomnia [11]. Further, withdrawal from caffeine is detectable overnight, and causes fatigue, stress, as well as decreased alertness and clear-headedness in heavy caffeine consumers [12,13,14].

The motivational desire to ingest a certain food incorporates a combination of flavor, learned associations, and physiological state that integrate to produce a food reward [15,16]. Since bitter taste is typically avoided by many species and may be an adaptation to protect them from adverse physiological effects, repeated consumption of caffeine may be a learned process [5]. The choice to consume caffeine may occur as a result of altered activation in brain areas related to reward pathways, particularly in areas associated with processing food rewards. Previous studies have reported that altered neuronal processing can occur as a consequence of repeated ingestion of a substance [17,18]. For example, habitual consumption of non-nutritive sweeteners has been associated with altered processing of sweet taste in individuals who regularly consume diet soda [17]. When compared to non-diet soda drinkers, diet soda drinkers demonstrated greater activation in areas related to reward processing, such as the dopaminergic midbrain, in response to sweet taste. Diet soda drinkers also exhibited greater activation in orbitofrontal cortex (OFC) Brodmann Area (BA) 47, an area related to pleasantness evaluation, when rating saccharin. Therefore, food consumption choices may be associated with altered neuronal activation.

The effects of caffeine consumption on central aspects of taste perception are not well understood. In addition to caffeine’s bitter taste [5], there is some suggestion from psychophysical studies that caffeine, which is an adenosine-receptor antagonist, may influence perception of some sweeteners through its action on adenosine receptors in sweet-sensitive taste cells [5,19,20]. The current study investigates differences between habitual caffeine consumers and non-consumers on brain activation during hedonic evaluation of taste, rather than the acute effects of caffeine consumption or withdrawal from caffeine consumption [21,22].

The purpose of the current study was to test the hypothesis that caffeine consumers and non-consumers may show differential brain activation, assessed with functional magnetic resonance imaging (fMRI), during hedonic evaluation of a bitter taste (caffeine), a sweet taste (sucrose), and a sweet taste with bitter after taste (saccharin). Results suggesting differential brain activation in association with caffeine consumption and different taste stimuli adds to preceding literature regarding caffeine’s influences on taste perception. Since caffeine consumption was a defining factor in group membership, it was chosen as the representation for bitter taste. Sweet taste was also chosen as a taste stimulus in response to preceding literature suggesting that caffeine may influence perception of sweet taste [5,19,20]. Saccharin was chosen as the third taste stimulus since it evokes a combination of bitter and sweet taste and may result in differential activation during taste processing in comparison to caffeine and/or sucrose. We aimed to investigate differential brain activation during the hedonic evaluation of taste to determine (1) whether caffeine consumers have greater activation than non-consumers in areas related to reward processing (e.g., nucleus accumbens, OFC BA 10); (2) whether caffeine non-consumers have greater neuronal activation than consumers in memory pathways, such as areas in the medial temporal lobe (MTL); and (3) whether caffeine non-consumers may rely upon activation of a larger network than consumers in order to perform the task.

## 2. Methods

### 2.1. Participants

The current sample (*n* = 28) consisted of 12 males and 16 females. Participants were divided into one of two groups: caffeine non-consumer (*n* = 14) and caffeine consumer (*n* = 14). Participants were divided into these groups based on answers to a survey that was administered after study completion. Participants who reported they not drink caffeinated beverages were labeled as caffeine non-consumers. Participants who responded that they did consume caffeinated beverages constituted the consumers group. Groups were matched on age, body mass index (BMI), and gender. Participants were part of a larger study investigating fMRI and taste processing. The Institutional Review Boards at San Diego State University and University of California, San Diego approved the study. All participants gave informed consent and were given monetary compensation for their participation.

### 2.2. Screening Session

The current study used the methodology described in detail in Haase, Cerf-Ducastel, Buracas, and Murphy (2007) [23]. All participants completed one screening session and one event-related fMRI session. At the initial screening, participant information, height, and weight were recorded. Participants were screened for metal in their body for the fMRI scan, as well as ageusia and anosmia with forced choice taste and odor threshold measures [24]. Being left-handed was an exclusionary criterion to avoid differential lateral activation in hemispheres due to handedness [25]. Participants who met the study criteria returned to complete one fMRI scan.

### 2.3. Odor and Taste Threshold Measures

In order to screen for anosmia, odor thresholds for the odor n-butyl alcohol (butanol) were assessed for each nostril monorhinically using a forced choice, ascending methods of limits test [24]. The solutions were in a series of 10; each dilution was one-third the concentration of the solution preceding it. On each trial the participant was presented with two bottles: one containing distilled water and the other containing the odor stimulus. The participant was asked to decide which bottle contained an odor. There was a 45 s inter-stimulus interval between each stimulus delivery to avoid adaptation [26]. If the participant chose the incorrect bottle, a higher concentration was given on the next trial. Once the participant met the criterion of choosing correctly on five successive trials the odor threshold was determined.

In order to screen for ageusia, taste thresholds for sucrose were assessed using a sip and spit, forced choice staircase procedure [24]. Stimuli were presented in 14 concentrations of sucrose, ranging from 0.0032 to 0.36 M in geometrical progression. All stimuli were presented at room temperature in distilled water [24]. The experimenter presented the participants with two cups, one containing distilled water and the other containing sucrose solution. The stimulus was sipped, held in the mouth for 10 s, and expectorated. After the participant sampled 10 ml of water and solution, he (she) was asked to select the stimulus with the sweet taste. The experimenter increased the concentration until the participant consistently (twice in a row) chose the stronger stimulus. This procedure was then reversed to a descending series until the participant failed to choose the correct stimulus. Participants were required to rinse with distilled water before each stimulus to avoid adaptation and waited a minimum of 30 s between each stimulus. Testing continued for five reversals with the mean of the last four reversals taken as the threshold.

### 2.4. Neuroimaging Procedure

Functional MRI data were collected in order to investigate brain response of caffeine consumer and caffeine non-consumer groups to stimuli during the physiological state of hunger. All scanning sessions occurred in the morning, and participants were instructed to fast 12 h prior to the scan. When stimuli were presented, participants used a joystick to rate pleasantness on a modified general Labeled Magnitude Scale. The scale was projected on a screen visible to the participant through a mirror attached to the head coil [23,27].

### 2.5. Stimulus Delivery

The stimuli used in this study were pure tastes delivered in aqueous solutions: 0.04 M caffeine, 0.64 M sucrose, and 0.028 M saccharin. These concentrations were chosen based on a previous study from our laboratory reporting how stimulus delivery method impacted the slopes of taste intensity functions for these stimuli [28]. The simulated stimulus delivery system was shown to produce psychophysical functions with slopes that were generally lower than experiments conducted with the sip and spit technique and that were similar to slopes of intensity functions associated with the dorsal flow procedure [28]. The concentrations chosen for the present study reflect the highest concentrations of each stimulus tested in Reference [28].

**Figure 1:**
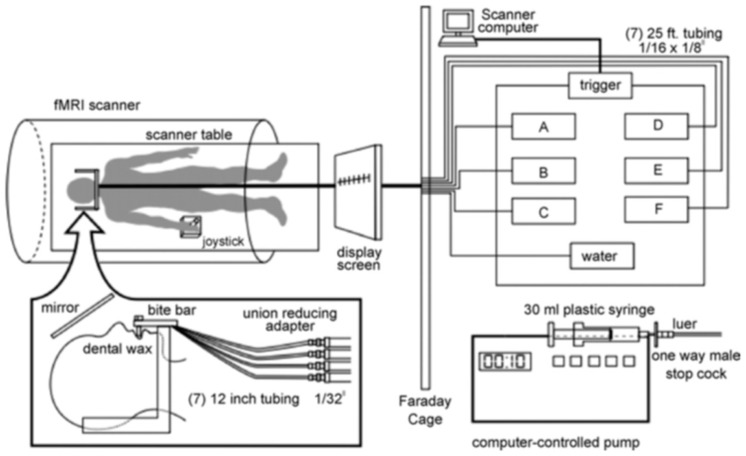
Stimulus delivery paradigm. Reprinted with permission from Haase et al. (2007) [23].

Stimuli were presented orally and presentations were randomized during functional data acquisition through the use of a computer-controlled delivery system (Figure 1). All taste stimuli were presented while the participant was inside the scanner, where the participant lay supine with a bite bar, which was positioned comfortably between the lips so that the tubes delivered stimuli to the tip of the tongue. Immediately before, during, and after the scan, participants rated the pleasantness and intensity of each stimulus. The taste stimuli and water were delivered at room temperature each through a unique 25-ft long plastic tube, which was connected to a different computer-programmable syringe pump. The pumps were programmed to present 0.3 mL of solution in 1 s.

The imaging session consisted of two functional runs. During the functional runs, each stimulus was presented in 0.3 mL of solution for a total of 16 times with a 10 s inter-stimulus interval. Participants were presented with water twice; first as a rinse, and then as a baseline to be used in data analysis. A complete outline of the stimulus delivery protocol used in the fMRI sessions is described in the Journal of Neuroscience Methods [23].

### 2.6. Imaging Acquisition

Functional MRI sessions took place at the Center for Functional Magnetic Resonance Imaging at the University of California, San Diego. All data were collected using a 3T General Electric Signa Excite short-bore scanner (GE Healthcare, Chicago, IL, USA). Structural data were acquired for anatomical localization of the functional images. Parameters used to acquire structural images were as follows: T1—weighted whole-brain fast spoiled gradient echo (FSPGR) sequences, field of view (FOV) = 25.6 cm, slice thickness = 1 mm, resolution = 1 × 1 × 1 mm^3^, echo time (TE) = 30 ms, Locs per slab = 190, flip angle = 15°. Parameters used to acquire functional images were as follows: T2*—weighted images, 32 axial slices, FOV = 19.2 cm, matrix size = 64 × 64, resolution = 3 × 3 × 3 mm^3^, flip angle = 90°, echo time (TE) = 30 ms, repetition time (TR) = 2000 ms.

### 2.7. Imaging Analysis

Imaging data were processed using FMRIB Software Library (FSL, Analysis Group, FMRIB, Oxford, UK) and Analysis of Functional NeuroImage (AFNI, open source software) [29,30]. Data were preprocessed to correct head movement and alignment as well as to concatenate the runs. Temporal and spatial smoothing of the brain images were also applied. Images were spatially smoothed to four full widths at half maximum (FWHM), auto-masked to remove voxels located outside of the brain, and normalized into Talairach space to control for individual variation in structural differences.

We conducted the analyses within AFNI, using 3dDeconvolve, on each participant’s concatenated runs based on the specified contrast (e.g., activation during evaluation of caffeine minus activation during evaluation of water) that accounted for the timing of delivery of the stimulus and the water baseline, which served as a control for identifying non-gustatory intra-oral stimulation [30,31]. Deconvolution estimates the hemodynamic response per voxel in a participant’s concatenated runs given the experimental paradigm (i.e., stimulus onset timing) using ordinary least squares regression. The output from 3dDeconvolve contains fit coefficients (i.e., beta weights) for each voxel, indicating the amplitude of the signal model for each contrast, and corresponding *t*-statistics.

Several thresholding steps were taken in an attempt to control for Type I error in all group analyses. Individual voxels were thresholded at *p* ≤ 0.015. To protect a whole-brain probability of false positives at an overall alpha of 0.05, group statistical maps were corrected for multiple comparisons at the cluster level using the AFNI program ClustSim [31]. ClustSim uses Monte Carlo simulations to compute the probability of generating a random “significant” cluster of noise (i.e., a false positive) given the individual voxel threshold, the voxel connection radius, the amount of blurring, and the search volume (i.e., overall dataset size). For an overall alpha level of 0.05, a cluster threshold of 21 contiguous voxels was applied. Neuronal activation in the caffeine consumers group during hedonic evaluation of the individual taste stimuli was subtracted from activation in the caffeine non-consumers group.

### 2.8. Demographic Data Analysis

To examine potential demographic differences between caffeine consumers and caffeine non-consumers, multivariate analyses of variance (MANOVA) were performed using caffeine status as an independent variable. Age, gender group, body mass index (BMI), taste threshold, right odor threshold, and left odor threshold were dependent variables. The results can be found in Table 1.

### 2.9. Psychophysical Data Analysis

The general Labeled Magnitude Scale (gLMS) was used to collect intensity ratings and a modified version of the gLMS was used to collect hedonic ratings for caffeine, sucrose, and saccharin taste before and after each scan [27]. To examine between group differences in psychophysical ratings, a MANOVA was performed using caffeine status as an independent variable. Results are shown in Table 2 and Table 3. Repeated measures analyses of variance (RM-ANOVA) were performed to examine possible differences between hedonic and intensity ratings of each taste before and after stimuli were presented during the scan.

## 3. Results

### 3.1. Demographic

There were no significant differences in age (F (1, 26) = 3.193, *p* = 0.086), BMI (F (1, 26) = 0.001, *p* = 0.972) or gender (F (1, 26) < 0.001, *p* = 1.000). There were also no significant differences in taste threshold (F (1, 26) = 0.169, *p* = 0.684) or in the odor threshold for the right nostril (F (1, 26) = 2.935, *p* = 0.099) or for the odor threshold for the left nostril (F (1, 26) = 1.252, *p* = 0.273).

### 3.2. Psychophysical Data

A MANOVA was performed to examine between group differences of hedonic and intensity ratings (Table 2 and Table 3). Caffeine non-consumers demonstrated significantly higher ratings for post-scan intensity ratings for sucrose (F (1, 26) = 4.390, *p* = 0.046) and saccharin (F (1, 26) = 7.312, *p* = 0.012) when compared to the post-scan intensity ratings for caffeine consumers.

There were no significant differences between caffeine consumers and non-consumers in pleasantness ratings of caffeine (F (1, 26) = 1.3686, *p* = 0.253), saccharin (F (1, 26) = 0.094, *p* = 0.762), or sucrose (F (1, 26) = 0.392 *p* = 0.537). There were also no significant differences between sucrose intensity ratings before and after stimuli were presented during the scan (F (1, 26) = 0.442, *p* = 0.512). There were significant differences between intensity ratings before and after the scan for caffeine (F (1, 26) = 10.173, *p* = 0.004) and saccharin (F (1, 26) = 6.558, *p* = 0.016). For caffeine consumers, neither saccharin intensity ratings (F (1, 13) = 0.568, *p* = 0.464) nor caffeine intensity ratings (F (1, 13) = 0.077, *p* = 0.786) were significantly different before and after taste was presented during the scan. For caffeine non-consumers, caffeine intensity ratings (F (1, 13) = 11.833, *p* = 0.004) and saccharin intensity ratings (F (1, 13) = 6.551, *p* = 0.024), were significantly different before and after the taste was presented. Intensity ratings for caffeine non-consumers were significantly higher after the stimuli were presented during the scan.

### 3.3. Functional Neuroimaging

**Figure 2:**
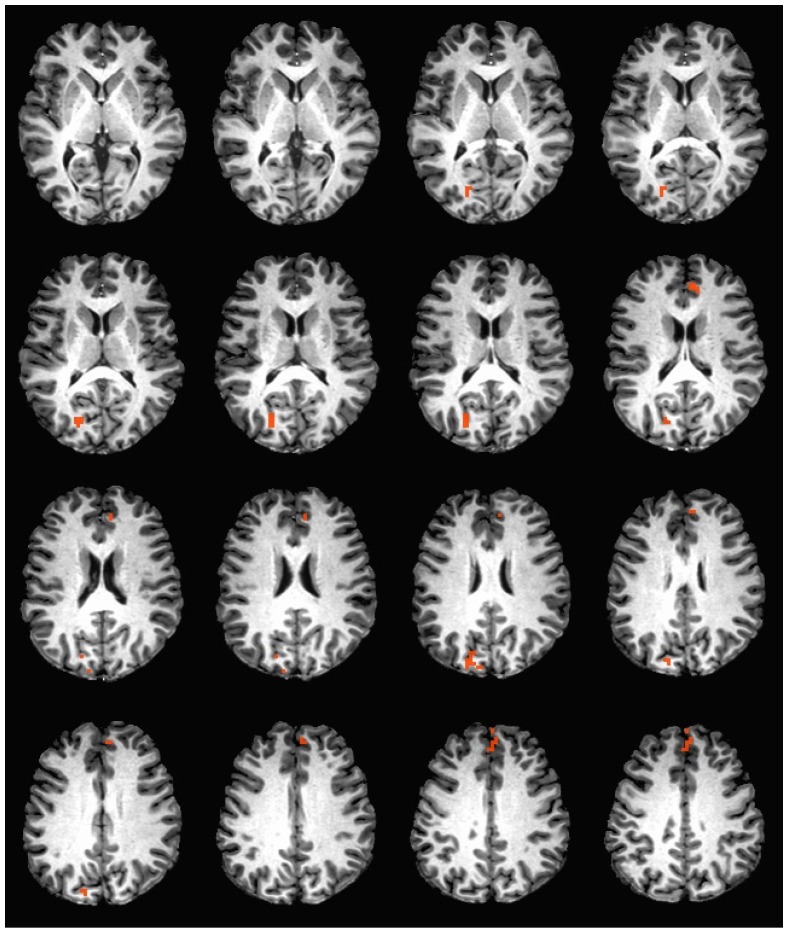
Brain activation during the hedonic evaluation of caffeine. Orange indicates areas where caffeine non-consumers had significantly greater activation in comparison to caffeine consumers.

During the hedonic evaluation of caffeine, caffeine non-consumers had significantly greater neuronal activation in the right cuneus, right precuneus, left anterior cingulate, medial frontal gyrus, and left superior frontal gyrus (See Table 4 and Figure 2).

**Figure 3:**
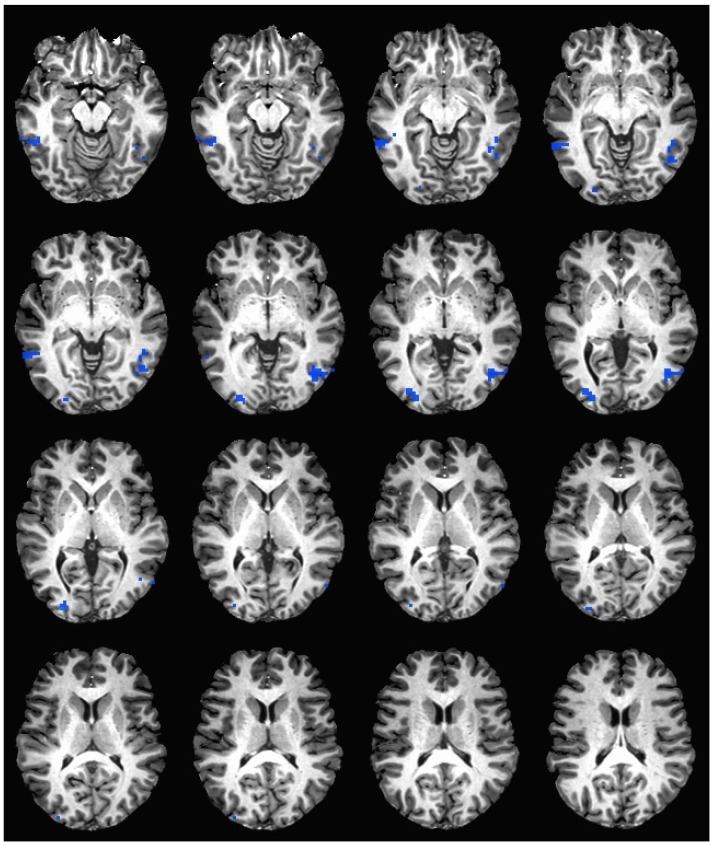
Brain activation during the hedonic evaluation of saccharin. Blue indicates areas where caffeine consumers had significantly greater activation in comparison to caffeine non-consumers.

During the hedonic evaluation of saccharin, caffeine non-consumers had significantly lower neuronal activation than caffeine consumers in the middle temporal gyrus, inferior temporal gyrus, middle occipital gyrus, right fusiform gyrus, right lingual gyrus, and right cuneus (See Table 5 and Figure 3).

**Figure 4:**
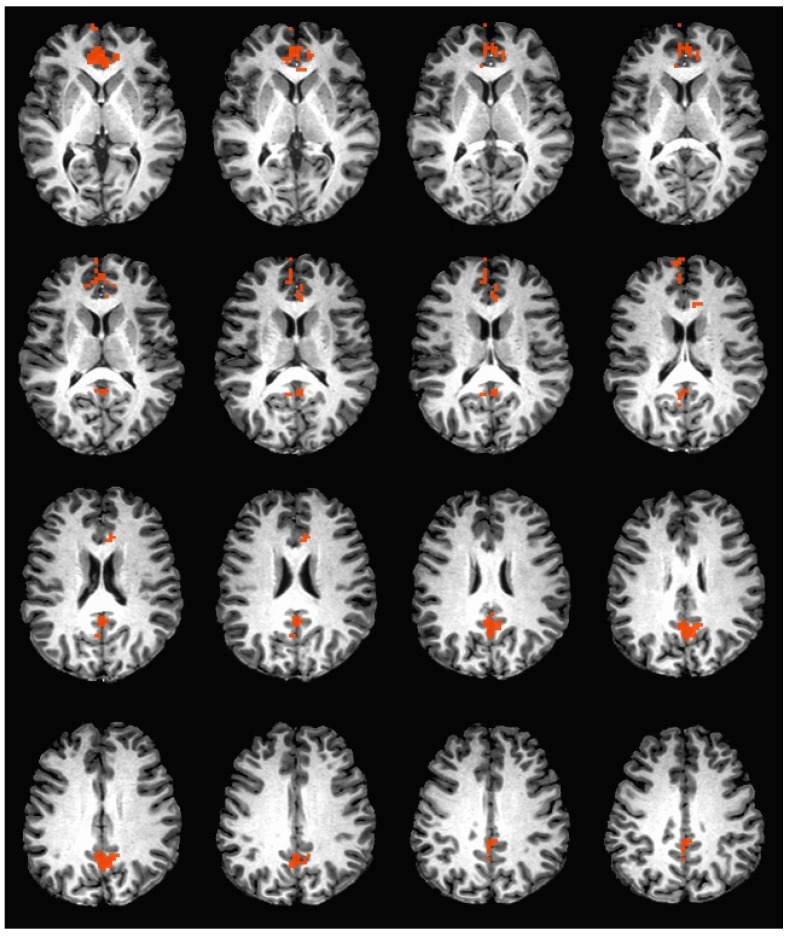
Brain activation during the hedonic evaluation of sucrose. Orange indicates areas where caffeine non-consumers had significantly greater activation in comparison to caffeine consumers.

During the hedonic evaluation of sucrose, caffeine non-consumers had significantly greater neuronal activation in the anterior cingulate, medial frontal gyrus, right superior frontal gyrus, OFC BA 10, posterior cingulate, cingulate gyrus, and precuneus (See Table 6 and Figure 4).

## 4. Discussion

How one perceives taste stimuli has been shown to influence food choice and repeated consumption of a tastant may lead to altered taste preferences [32,33,34,35]. There are neuroimaging data to suggest that the human brain responds differently as a result of habitual consumption [17,18]. However, to our knowledge, there is no human research investigating brain response during hedonic evaluation of taste in caffeine consumers and non-consumers. In this study, we examined brain response in self-reported caffeine consumers and caffeine non-consumers during an fMRI scan to investigate whether regular consumption of caffeine is associated with differential activation of areas related to memory, reward, and information processing. Imaging data from the present study indicate that caffeine consumers and caffeine non-consumers have significantly different neuronal activations in areas related to memory, reward, and information processing when processing individual taste stimuli. Each participant was exposed to 0.3 mL/sec of each tastant for 16 repetitions resulting in a total consumption of <5 mL, suggesting that these differences in activation occurred as a result from processing the taste alone, rather than the possible physiological effects of ingestion. When rating caffeine and sucrose, caffeine non-consumers had significantly greater activation in areas related to memory, reward, and information processing. During hedonic evaluation of saccharin, caffeine consumers had significantly greater activation in areas related to information processing. Overall, our results indicate differential neuronal activations between both groups during the processing of all three tastes. These results suggest differences in overall cognitive expenditure between the two groups, differing based on which taste was presented.

### 4.1. Psychophysical Data

Caffeine consumers and caffeine non-consumers demonstrated differences in taste perception. Post-scan intensity ratings of sucrose and saccharin were significantly higher in caffeine non-consumers compared to caffeine consumers. Further, caffeine non-consumer ratings of caffeine and sucrose intensity significantly increased from before to after stimulus presentation within an fMRI scan. The latter phenomena were not present in caffeine consumers.

Psychophysical results suggest that caffeine non-consumers perceived sucrose and saccharin as being more intense than caffeine consumers after the scan. Also, caffeine and saccharin intensity ratings significantly increased after stimuli were presented during the scan for caffeine non-consumers. A plausible explanation is that the sweet taste of sucrose and saccharin may have been potentiated by caffeine [20]. The increase of perceived intensity of caffeine and saccharin after the scans in non-consumers suggests a stronger reaction to bitter taste, which is present in caffeine and in saccharin as an aftertaste. There is evidence that the perceived intensity of caffeine’s bitterness may be associated with whether caffeine is regularly consumed and the expression of bitter receptors, PAV-TAS2R38 [5,36]. It is plausible that both a genetic predisposition and caffeine consumption habits contributed to caffeine non-consumers perceiving all three tastes more intensely in comparison to caffeine consumers.

### 4.2. Reward Processing Areas

During the hedonic evaluation of caffeine and sucrose, caffeine non-consumers demonstrated greater activation in areas associated with reward processing.

During the hedonic evaluation of sucrose, caffeine non-consumers demonstrated significantly greater activation in both hemispheres of OFC BA 10, an area associated with encoding the incentive value of a stimulus during a decision-making task [37,38,39]. The OFC has been activated in response to abstract internal goals, such as rewards and punishments, while other tasks are being performed [37,38,39]. The OFC has been reported to be responsive to the reward value of tastes, as it associates other stimuli with tastes to produce representations of expected reward value [37,40]. A reward stimulus has been found to induce increased activation in OFC BA 10 when already activated by working memory processing [41]. Further, the OFC is activated by monetary rewards and punishment, with more activation reported following a punishment outcome [38].

During the hedonic evaluation of caffeine and sucrose, activation in the anterior cingulate cortex (ACC) was significantly greater in caffeine non-consumers. During the hedonic evaluation of caffeine, only activation in the left anterior cingulate cortex was found to be significantly greater in caffeine non-consumers in comparison to caffeine consumers. Lateralization in the ACC has been found during error processing and conflict monitoring, where correct inhibitions only occurred in the right ACC [42]. Further, observational fear learning has been found to only be activated in the right, but not the left ACC [43]. The distinction that right ACC activation only occurred during the hedonic evaluation of sucrose and not during the hedonic evaluation of caffeine suggests that sucrose may have been a more intense experience for caffeine non-consumers. Psychophysical data supports this assertion, as caffeine non-consumers provided significantly higher intensity ratings for sucrose post-scan when compared to caffeine consumers (Table 3).

Overall, the ACC has been associated with an overall neural circuit that uses past action-reward history to learn action value in order to guide voluntary choice behavior [44]. This process requires referencing a history of outcomes regarding a given choice [44]. Further, previous studies suggest that reward processing in the ACC may also guide choice behavior, as it relates actions to their consequences [45]. This suggests that ACC has an essential role in learning and using extended action-outcome histories to make voluntary choices.

It is important to emphasize that activity in the OFC is representative not merely of a reward per se, but of a detailed and information rich representation of reward [46]. Similarly, the ACC references past-action reward history and is not a direct reflection of the reward value [44,45]. Therefore, the results are not necessarily indicative of caffeine non-consumers finding tastes to be more or less rewarding than caffeine consumers. A more plausible explanation may be that greater activation in the OFC and ACC found in caffeine non-consumers suggests a greater cognitive expenditure to use past reward history and process the representation of a reward, in order to make a voluntary choice, which in this case, was the hedonic rating.

### 4.3. Memory Processing Areas

During the hedonic evaluation of caffeine, caffeine non-consumers demonstrated significantly greater activation in right precuneus. During the hedonic evaluation of sucrose, caffeine non-consumers demonstrated significantly greater activation in both the left and right side of the precuneus. The right precuneus has been previously linked to autobiographical memory retrieval [47]. It is of particular interest that this area was activated during the hedonic evaluation of caffeine, an experience that would not be common in caffeine non-consumers. The precuneus is an area previously associated with episodic memory retrieval, the ability to recall a previously experienced stimulus [48]. Continuous theta burst stimulation (cTBS) over the precuneus in a picture memory task was associated with a decrease in source memory errors and improvement in context retrieval, suggesting that the precuneus is integral to a memory encoding and retrieval network [48]. During a source and item-recognition memory task, the left precuneus was activated during memory retrieval [49].

During the evaluation of sucrose, caffeine non-consumers also demonstrated greater activation in both the left and right of the posterior cingulate and cingulate gyrus. The posterior cingulate cortex has been associated with memory retrieval, namely autobiographical memory retrieval [50]. The posterior cingulate cortex also subserves evaluative functions such as monitoring sensory events and behavioral actions in the service of spatial orientation and memory [51].

These results support the hypothesis that caffeine non-consumers demonstrate greater cognitive expenditure in memory processing areas. We speculate that greater activations in the caffeine non-consumers while evaluating caffeine and sucrose could indicate a greater source memory retrieval expenditure. It is possible that caffeine non-consumers may have had less exposure to these tastes due to their dietary choices, and therefore, require greater cognitive effort to process them. Further, while sucrose is ubiquitous in all types of food, it is possible that experiencing caffeine’s bitter taste is a new experience for caffeine non-consumers, not only in experiencing caffeine’s flavor profile, but also its subsequent impact on other tastes.

### 4.4. Information Processing

Activation in information processing pathways was observed during hedonic evaluation of all three tastants. Activation in the right superior frontal gyrus (SFG) was significantly higher during the hedonic evaluation of sucrose. The right SFG has been linked to functioning in cognitive control, such that greater activation was linked to more efficient response inhibition, less motor urgency, as well as greater self-regulation [52,53]. The left SFG was significantly higher during the hedonic evaluation of caffeine in caffeine non-consumers. The superior frontal gyrus, particularly the left SFG, has been associated with performing higher cognitive functions associated with working memory retrieval, especially in relation to task-related behavioral goals [54].

Both sides of the medial frontal gyrus were significantly activated in caffeine non-consumers during the hedonic evaluation of caffeine and sucrose, but not in the saccharin condition. Previous studies have linked activation in the left dorsolateral prefrontal cortex to processing and rating multimodal flavor stimuli [55]. Further, this is an area where the consequences of actions directly affect cognition in the preparation for and selection of response [55]. Results suggest a greater cognitive effort during the hedonic evaluation of caffeine and sucrose for caffeine non-consumers and during saccharin for caffeine consumers in information processing pathways coinciding with results previously stated. Due to the variability in between group activation within information processing areas, it is difficult to make a conclusive decision whether or not caffeine non-consumers activate a larger network than consumers in order to perform the hedonic evaluation task. While there was primarily more activation within the overall study in caffeine non-consumers, caffeine consumers demonstrated greater activation during the saccharin condition. We speculate that greater activation for caffeine consumers during saccharin evaluation may have occurred because saccharin evokes both sweet and bitter taste [56]. In addition to the stimulation of both sweet and bitter receptors, additional expenditure of cognitive effort may be required to hedonically evaluate this taste experience.

### 4.5. Further Considerations

There are limitations to this study. We did not investigate the potential differences in response between caffeine consumers who regularly consume caffeinated beverages with a higher sugar content and caffeine consumers who more regularly consume more bitter tasting beverages. Future studies may differentiate between the impact of taste processing for habitual consumers that drink primarily bitter tasting beverages (i.e., black coffee and tea) or items greater in sugar content (i.e., energy drinks). Further, in the caffeine consumers group, there were varying levels of caffeine consumption. Future studies may choose to expand on this paradigm, considering the effect of varying types and levels of caffeine consumption on taste perception.

We did not specify whether the taste stimuli were administered to the left or right side of the tongue. While we could not locate literature detailing a lateralization in processing of sweet and bitter taste alone, previous studies have reported laterization when discriminating tastes and rating taste quality [57,58]. Stevenson, Miller, and McGrillen [58] reported that when administering sour, sweet, salty, bitter, and umami solutions, discrimination among tastes was better when stimuli were applied to the right tongue tip and participants were better at taste quality judgements when tastants were applied to the left tongue tip [58]. All stimuli in the present study were administered to the tip of the tongue and whether the stimuli were more exposed to the left or the right side on trials was not specified. However, future studies could elaborate on this paradigm by taking this lateralization of gustatory processing into account.

The effects of caffeine consumption on taste perception are of considerable interest. Following a report that adenosine can enhance sweet taste in mice through its actions on A2B receptors in the taste bud, a recent report of a human psychophysical study suggested that caffeine, which is an adenosine-receptor antagonist, may decrease the perceived intensity of sweet taste through its action on adenosine receptors in sweet-sensitive taste cells [19,59]. Early studies of the effects of caffeine on taste had reported that in aqueous solutions of two component mixtures, caffeine decreased the sweetness of sucrose; and that when applied directly to the tongue with filter paper, caffeine enhanced the intensity of quinine HCl, NaCl, and a number of nonnutritive sweeteners, particularly those with bitter components (e.g., saccharin), but not the nutritive sweeteners sucrose and fructose [20,60]. The acute ingestion of caffeine has been reported to reduce the intensity of saccharin but not other taste stimuli, and that raising caffeine levels in the saliva for a period of three weeks had no measurable effects on reported intensity of caffeine, denatonium benzoate or NaCl [61,62]. Differences in the effects of caffeine on sweetness intensity may be related to the stimuli, their concentrations, the route of administration or other methodological differences in these studies [19,20,60,61,62]. The current study focused on the effects of habitual caffeine consumption on fMRI of central brain response and found differential activation between caffeine consumers and non-consumers during hedonic evaluation of sucrose, caffeine, and saccharin stimuli. Further research on both intensity and hedonics of bitter and sweet stimuli, including natural as well as artificial sweeteners, in caffeine consumers and non-consumers will be of great interest to better understand the nature of caffeine’s influence on taste perception.

## 5. Conclusions

In summary, we administered three tastants, caffeine, sucrose, and saccharin, to investigate differences in neuronal activation between those who were self-reported caffeine consumers and caffeine non-consumers. We found differences in intensity ratings between groups. We also found differences in activation patterns during a hedonic evaluation task. Our results suggest that there is greater activation for caffeine non-consumers while processing caffeine and sucrose and greater activation for caffeine consumers while rating saccharin. The results support differential memory, reward, and information processing of taste between those who habitually consume caffeine and those who do not. These results suggest that further research into the link between caffeine consumption and taste perception is warranted.

## Figures and Tables

**Table 1:** Participant characteristics for caffeine non-consumers and matched caffeine consumers.

	Caffeine Non-Consumers		Caffeine Consumers			
Demographics	Mean	SD	Mean	SD	F	*p*
Age	56.786	15.837	45.00	18.925	3.193	0.086
Gender	0.571	0.514	0.571	0.514	0.000	1.000
BMI	29.564	6.783	29.654	6.208	0.001	0.972
Taste Threshold	0.005	0.005	0.006	0.011	0.169	0.684
Odor Threshold R	6.000	1.797	7.000	1.240	2.935	0.099
Odor Threshold L	6.140	1.875	6.786	1.051	1.252	0.273

BMI: body mass index.

**Table 2:** Hedonic ratings for caffeine non-consumers and matched caffeine consumers.

	Caffeine Non-Consumers		Caffeine Consumers			
Hedonic Ratings	Mean	SD	Mean	SD	F	*p*
Caffeine Pre	35.286	16.973	39.357	15.619	0.436	0.515
Caffeine Post	26.357	16.284	38.214	14.766	4.073	0.054
Sucrose Pre	58.786	11.943	61.929	10.095	0.565	0.459
Sucrose Post	52.929	18.512	60.500	9.053	1.890	0.181
Saccharin Pre	51.429	15.500	55.357	7.938	0.712	0.406
Saccharin Post	47.429	18.793	53.360	9.740	1.098	0.304

**Table 3:** Intensity ratings for caffeine non-consumers and matched caffeine consumers.

	Caffeine Non-Consumers		Caffeine Consumers			
Intensity Ratings	Mean	SD	Mean	SD	F	*p*
Caffeine Pre	29.429	20.470	35.929	27.280	0.508	0.482
Caffeine Post	53.786	31.499	34.929	24.656	3.111	0.090
Sucrose Pre	41.786	24.974	28.071	12.982	3.324	0.080
Sucrose Post *	52.000	30.894	32.929	14.334	4.390	0.046
Saccharin Pre	35.071	23.206	34.214	16.348	0.013	0.911
Saccharin Post *	52.357	26.401	31.000	13.278	7.312	0.012

* Significant difference between caffeine consumers and caffeine non-consumers.

**Table 4:** Regions of significantly greater activity in caffeine non-consumers compared to caffeine consumers while judging the pleasantness of caffeine.

		Talaraich Coordinates				
Region	Hem.	X	Y	Z	Regr. Coef.	Voxels in Cluster
Cuneus	R	8	−85	26	1.76	38
Precuneus	R	16	−74	26	0.33	
Medial Frontal Gyrus	L	−1	47	41	0.757	28
Medial Frontal Gyrus	R	0	45	39	0.608	
Superior Frontal Gyrus	L	−1	54	34	0.65	
Anterior Cingulate	L	−7	38	22	0.52	

Hem.: Hemisphere; R: right; L: left; Regr. Coef.: Regression coefficient; Minimum cluster = 21 voxels, *p* = 0.015.

**Table 5:** Regions of significantly greater activity in caffeine consumers compared to caffeine non-consumers while judging the pleasantness of saccharin.

		Talairach Coordinates				
Region	Hem.	X	Y	Z	Regr. Coef.	Voxels in Cluster
Middle Temporal Gyrus	L	−55	−64	5	−0.87	50
Inferior Temporal Gyrus	L	−44	−69	−1	−0.617	
Middle Occipital Gyrus	L	−44	−62	−3	−0.396	
Middle Temporal Gyrus	R	59	−46	−7	−0.647	28
Inferior Temporal Gyrus	R	63	−48	−7	−0.39	
Fusiform Gyrus	R	46	−38	−7	−0.289	
Middle Occipital Gyrus	R	29	−88	5	−1.12	27
Lingual Gyrus	R	27	−89	−2	−0.849	
Cuneus	R	23	−91	−1	−0.712	

Hem.: Hemisphere; R: right; L: left; Regr. Coef.: Regression coefficient; Minimum cluster = 21 voxels, *p* = 0.015.

**Table 6:** Regions of significantly greater activity in caffeine non-consumers compared to caffeine consumers while judging the pleasantness of sucrose.

		Talairach Coordinates				
Region	Hem.	X	Y	Z	Regr. Coef.	Voxels in Cluster
Anterior Cingulate	R	2	41	−1	1.26	153
Medial Frontal Gyrus	R	2	62	20	1.03	
Anterior Cingulate	L	−2	42	−1	0.937	
OFC BA10	R	5	62	14	0.63	
Superior Frontal Gyrus	R	10	59	21	0.535	
Medial Frontal Gyrus	L	−10	40	14	0.446	
OFC BA10	L	−10	43	12	0.421	
Posterior Cingulate	L	−1	−46	14	1.27	90
Posterior Cingulate	R	2	47	13	1.24	
Cingulate Gyrus	R	2	−49	27	0.862	
Cingulate Gyrus	L	0	−50	29	0.64	
Precuneus	R	2	−49	32	0.583	
Precuneus	L	−7	−58	29	0.537	

Hem.: Hemisphere; R: right; L: left; Regr. Coef.: Regression coefficient; Minimum cluster = 21 voxels, *p* = 0.015.

## References

[1] Cappelletti S., Daria P., Sani G., Aromatario M. (2015). Caffeine: Cognitive and Physical Performance Enhancer or Psychoactive Drug?. Curr. Neuropharmacol..

[2] Mitchell D.C., Knight C.A., Hockenberry J., Teplansky R., Hartman T.J. (2014). Beverage caffeine intakes in the U.S. Food Chem. Toxicol..

[3] Smith J.E., Lawrence A.D., Diukova A., Wise R.G., Rogers P.J. (2012). Storm in a coffee cup: Caffeine modifies brain activation to social signals of threat. Soc. Cogn. Affect. Neurosci..

[4] Clauson K.A., Shields K.M., McQueen C.E., Persad N. (2008). Safety issues associated with commercially available energy drinks. J. Am. Pharm. Assoc..

[5] Poole R.L., Tordoff M.G. (2017). The Taste of Caffeine. J. Caffeine Res..

[6] Glade M.J. (2010). Caffeine—Not just a stimulant. Nutrition.

[7] Rao A., Henglong H., Nobre A.C. (2005). The effects of combined caffeine and glucose drinks on attention in the human brain. Nutr. Neurosci..

[8] Quinlan P.T., Lane J., Moore K.L., Aspen J., Rycroft J.A., O’Brien D.C. (2000). The Acute Physiological and Mood Effects of Tea and Coffee: The Role of Caffeine Level. Pharmacol. Biochem. Behav..

[9] Park C.A., Kang C.K., Son Y.D., Choi E.J., Kim S.H., Oh S.T., Kim Y.B., Park C.W., Cho Z.H. (2014). The effects of caffeine ingestion on cortical areas: Functional imaging study. Magnet. Reson. Imaging.

[10] Van Schouwenburg M.R., den Ouden H.E.M., Cools R. (2010). The Human Basal Ganglia Modulate Frontal-Posterior Connectivity during Attention Shifting. J. Neurosci..

[11] Nehlig A., Boyet S. (2000). Dose–response study of caffeine effects on cerebral functional activity with a specific focus on dependence. Brain Res..

[12] Rogers P.J., Hohoff C., Heatherley S.V., Mullings E.L., Maxfield P.J., Evershed R.P., Deckert J., Nutt D.J. (2010). Association of the Anxiogenic and Alerting Effects of Caffeine with ADORA2A and ADORA1 Polymorphisms and Habitual Level of Caffeine Consumption. Neuropsychopharmacology.

[13] Ratliff-Crain J., O’Keeffe M.K., Baum A. (1989). Cardiovascular reactivity, mood, and task performance in deprived and nondeprived coffee drinkers. Health Psychol..

[14] Schuh K.J., Griffiths R.R. (1997). Caffeine reinforcement: The role of withdrawal. Psychopharmacology.

[15] Berridge K.C. (1996). Food reward: Brain substrates of wanting and liking. Neurosci. Biobehav. Rev..

[16] Rogers P.J., Hardman C.A. (2015). Food reward. What it is and how to measure it. Appetite.

[17] Green E., Murphy C. (2012). Altered processing of sweet taste in the brain of diet soda drinkers. Physiol. Behav..

[18] Green E., Jacobson A., Haase L., Murphy C. (2011). Reduced nucleus accumbens and caudate nucleus activation to a pleasant taste is associated with obesity in older adults. Brain Res..

[19] Choo E., Picket B., Dando R. (2017). Caffeine May Reduce Perceived Sweet Taste in Humans, Supporting Evidence That Adenosine Receptors Modulate Taste. J. Food Sci..

[20] Schiffman S.S., Diaz C., Beeker T.G. (1986). Caffeine intensifies taste of certain sweeteners: Role of adenosine receptor. Pharmacol. Biochem. Behav..

[21] Field A.S., Laurienti P.J., Yen Y.-F., Burdette J.H., Moody D.M. (2003). Dietary Caffeine Consumption and Withdrawal: Confounding Variables in Quantitative Cerebral Perfusion Studies?. Radiology.

[22] Laurienti P.J., Field A.S., Burdette J.H., Maldjian J.A., Yen Y.-F., Moody D.M. (2002). Dietary Caffeine Consumption Modulates fMRI Measures. NeuroImage.

[23] Haase L., Cerf-Ducastel B., Buracas G., Murphy C. (2007). On-line psychophysical data acquisition and event-related fMRI protocol optimized for the investigation of brain activation in response to gustatory stimuli. J. Neurosci. Methods.

[24] Murphy C., Gilmore M.M., Seery C.S., Salmon D.P., Lasker B.R. (1990). Olfactory thresholds are associated with degree of dementia in Alzheimer’s disease. Neurobiol. Aging.

[25] Royet J.P., Plailly J., Delon-Martin C., Kareken D.A., Segebarth C. (2003). fMRI of emotional responses to odors: Influence of hedonic valence and judgment, handedness, and gender. NeuroImage.

[26] Ekman G., Berglund B., Berglund U., Lindvall T. (1967). Perceived intensity of odor as a function of time of adaptation. Scand. J. Psychol..

[27] Bartoshuk L.M., Duffy V.B., Green B.G., Hoffman H.J., Ko C.W., Lucchina L.A., Marks L.E., Snyder D.J., Weiffenbach J.M. (2004). Valid across-group comparisons with labeled scales: The gLMS versus magnitude matching. Physiol. Behav..

[28] Haase L., Cerf-Ducastel B., Murphy C. (2009). The effect of stimulus delivery technique on perceived intensity functions for taste stimuli: Implications for fMRI studies. Atten. Percept. Psychophys..

[29] Smith S.M., Jenkinson M., Woolrich M.W., Beckman C.F., Behrens T.E.J., Johansen-Berg H., Bannister P.R., De Luca M., Drobnjak I., Flitney D.E. (2004). Advances in functional and structural MR image analysis and implementation as FSL. NeuroImage.

[30] Cox R.W. (1996). AFNI: Software for Analysis and Visualization of Functional Magnetic Resonance Neuroimages. Comput. Biomed. Res..

[31] Zald D.H., Pardo J.V. (2000). Functional neuroimaging of the olfactory system in humans. Int. J. Psychophysiol..

[32] Nolden A.A., Hayes J.E. (2015). Perceptual Qualities of Ethanol Depend on Concentration, and Variation in These Percepts Associates with Drinking Frequency. Chemosens. Percept..

[33] Zandstra E.H., De Graaf C., Mela D.J., Van Staveren W.A. (2000). Short- and long-term effects of changes in pleasantness on food intake. Appetite.

[34] Bolhuis D.P., Gijsbers L., de Jager I., Geleijnse J.M., de Graaf K. (2015). Encapsulated sodium supplementation of 4 weeks does not alter salt taste preferences in a controlled low sodium and low potassium diet. Food Qual. Prefer..

[35] Bertino M., Beauchamp G.K., Engelman K. (1982). Long-term reduction in dietary sodium alters the taste of salt. Am. J. Clin. Nutr..

[36] Lipchock S.V., Spielman A.I., Mennella J.A., Mansfield C.J., Hwang L.-D., Douglas J.E., Reed D.R. (2017). Caffeine Bitterness is Related to Daily Caffeine Intake and Bitter Receptor mRNA Abundance in Human Taste Tissue. Perception.

[37] Simmons W.K., Martin A., Barsalou L.W. (2005). Pictures of Appetizing Foods Activate Gustatory Cortices for Taste and Reward. Cerebr. Cortex.

[38] O’Doherty J., Kringelbach M.L., Rolls E.T., Hornak J., Andrews C. (2001). Abstract reward and punishment representations in the human orbitofrontal cortex. Nat. Neurosci..

[39] Green E., Jacobson A., Haase L., Murphy C. (2015). Neural correlates of taste and pleasantness evaluation in the metabolic syndrome. Brain Res..

[40] Rolls E.T. (2017). The orbitofrontal cortex and emotion in health and disease, including depression. Neuropsychologia.

[41] Pochon J.B., Levy R., Fossati P., Lehericy S., Poline J.B., Pillon B., Le Bihan D., Dubois B. (2002). The neural system that bridges reward and cognition in humans: An fMRI study. Proc. Natl. Acad. Sci. USA.

[42] Lütcke H., Frahm J. (2008). Lateralized Anterior Cingulate Function during Error Processing and Conflict Monitoring as Revealed by High-Resolution fMRI. Cereb. Cortex.

[43] Kim S., Mátyás F., Lee S., Acsády L., Shin H.-S. (2012). Lateralization of observational fear learning at the cortical but not thalamic level in mice. Proc. Natl. Acad. Sci. USA.

[44] Kennerley S.W., Walton M.E., Behrens T.E.J., Buckley M.J., Rushworth M.F.S. (2006). Optimal decision making and the anterior cingulate cortex. Nat. Neurosci..

[45] Rushworth M., Walton M., Kennerley S., Bannerman D. (2004). Action sets and decisions in the medial frontal cortex. Trends Cogn. Sci..

[46] Rolls E.T. (2000). The Orbitofrontal Cortex and Reward. Cereb. Cortex.

[47] Freton M., Lemogne C., Bergouignan L., Delaveau P., Lehéricy S., Fossati P. (2014). The eye of the self: Precuneus volume and visual perspective during autobiographical memory retrieval. Brain Struct. Funct..

[48] Bonnì S., Veniero D., Mastropasqua C., Ponzo V., Caltagirone C., Bozzali M., Koch G. (2015). TMS evidence for a selective role of the precuneus in source memory retrieval. Behav. Brain Res..

[49] Lundstrom B., Petersson K.M., Andersson J., Johansson M., Fransson P., Ingvar M. (2003). Isolating the retrieval of imagined pictures during episodic memory: Activation of the left precuneus and left prefrontal cortex. NeuroImage.

[50] Maddock R.J., Garrett A.S., Buonocore M.H. (2001). Remembering familiar people: The posterior cingulate cortex and autobiographical memory retrieval. Neuroscience.

[51] Vogt B.A., Finch D.M., Olson C.R. (1992). Functional Heterogeneity in Cingulate Cortex: The Anterior Executive and Posterior Evaluative Regions. Cereb. Cortex.

[52] Hu S., Ide J.S., Zhang S., Li C.R. (2016). The Right Superior Frontal Gyrus and Individual Variation in Proactive Control of Impulsive Response. J. Neurosci..

[53] Beauregard M., Lévesque J., Bourgouin P. (2001). Neural Correlates of Conscious Self-Regulation of Emotion. J. Neurosci..

[54] Boisgueheneuc F.D., Levy R., Volle E., Seassau M., Duffau H., Kinkingnehun S., Samson Y., Zhang S., Dubois B. (2006). Functions of the left superior frontal gyrus in humans: A lesion study. Brain.

[55] Kringelbach M.L., de Araujo I.E., Rolls E.T. (2004). Taste-related activity in the human dorsolateral prefrontal cortex. NeuroImage.

[56] Lawless H. (1982). Adapting efficiency of salt-sucrose mixtures. Percept. Psychophys..

[57] Prinster A., Cantone E., Verlezza V., Magliulo M., Sarnelli G., Iengo M., Cuomo R., Di Salle F., Esposito F. (2017). Cortical representation of different taste modalities on the gustatory cortex: A pilot study. PLoS ONE.

[58] Stevenson R.J., Miller L.A., McGrillen K. (2013). The lateralization of gustatory function and the flow of information from tongue to cortex. Neuropsychologia.

[59] Dando R., Dvoryanchikov G., Pereira E., Chaudhari N., Roper S.D. (2012). Adenosine enhances sweet taste through A2B receptors in the taste bud. J. Neurosci..

[60] Pangborn R. (1960). Taste interrelationships. J. Food Sci..

[61] Mela D.J. (1989). Caffeine ingested under natural conditions does not alter taste intensity. Pharmacol. Biochem. Behav..

[62] Mela D.J., Mattes R.D., Tanimura S., Garcia-Medina M.R. (1992). Relationships between ingestion and gustatory perception of caffeine. Pharmacol. Biochem. Behav..

